# The double role of pigmentation and convolute leaves in community assemblage of Amazonian epiphytic Lejeuneaceae

**DOI:** 10.7717/peerj.5921

**Published:** 2018-11-30

**Authors:** Sylvia Mota de Oliveira

**Affiliations:** Biodiversity Discovery Group, Naturalis Biodiversity Center, Leiden, The Netherlands

**Keywords:** Amazonia, Bryophyte, Community assembly, Habitat specialization, Species sorting

## Abstract

**Background:**

Epiphytic bryophyte communities in the Amazon forest show a vertical gradient in species composition along the trunk of the host trees. The investigation of species traits related to this pattern has focused on the physiology of selected taxa with a clear preference for one of the extremes of the gradient. Although some species are indeed only found on the tree base or in the outer canopy, the vertical gradient is composed mainly by the variation in the abundances of species with a broader occurrence along the height zones. Therefore, this study approaches the differences among community assemblages, rather than among species, to test the role of morphological and dispersal traits on the establishment of the vertical gradient in species composition.

**Methods:**

A character state matrix was built for 104 species of the family Lejeuneaceae recorded as epiphytes in the Amazonian terra firme forests, and six binary traits supposed to influence species occurrence: dark pigmentation on leaves; ability to convolute leaves when drying; possession of thickened cell walls; reproduction mode (monoicous or dioicous); occurrence of asexual reproduction; and facultative epiphyllous habit. Based on a previous dataset on community composition along the vertical gradient, trait occurrences in random draws of the metacommunity was compared to trait occurrences in field data, in order to detect significant deviations in the different height zones.

**Results:**

Four out of the six traits tested showed significantly higher or lower occurrence in the species composition of canopy and/or understory communities. Traits related to high dispersal ability did not vary much along the vertical gradient; although facultative epiphylls were overrepresented on tree base. Dark pigmentation and convolute leaves were significantly more frequent in the canopy communities, but also significantly less frequent in communities at the base of the tree.

**Discussion:**

Dark pigmentation and convolute leaves seem to be advantageous for the establishment in the canopy zones. They may, respectively, prevent light damage and allow longer periods of photosynthesis. Interestingly, these traits occur randomly along the trunk, but are wiped out of communities on the tree base. In the relatively deep shade of the first meters of the understory, they possibly hamper net carbon gain, the first by darkening the leaf surface and the second by delaying desiccation—which can be damaging under high temperatures and low light. The fact that production of asexual propagules is not overrepresented in the most dynamic microenvironment along the gradient, the canopy, challenges current views of bryophyte life strategy theory.

## Introduction

About a century ago, W. Watson published one of the first extensive discussions on the bryophyte morphological features that could be related to species occurrence ( Watson, 1914), starting with: “*when examining the bryophytes of the woodlands of Somersetshire (…), I was desirous of obtaining information on the characters which enable some species to live in dry situations, whilst others could only exist when provided with a large amount of shade or moisture”*. Watson’s implicit assumption of niche assembly—in the broad sense that species features have a role on species occurrence—as well as his emphasis on environmental filtering, rather than on species interactions, illustrates the common sense in bryophyte ecology (see also Slack, 1990). Accordingly, studies on the role of species features on bryophyte occurrence have been concentrated on a number of selected species that demonstrate a clear preference for contrasting environmental conditions (Gabriel & Bates, 2003; Rincon & Grime, 1989; Tobiessen, Slack & Mott, 1979). The fact that the species studied are selected *because* they have a strong environmental preference prevents one to conclude how far the investigated character actually influences the establishment of the ecological gradient, usually formed by a much higher number of species than the ones tested. Analyses that make use of the concept of metacommunity, under a species sorting framework (Leibold et al., 2004), allow testing whether species traits play a role in the process of community assemblage (Wang et al., 2017), an approach more frequently used for vascular plants (Kraft, Valencia & Ackerly, 2008; Lebrija-Trejos et al., 2010).

Epiphytic bryophyte communities in tropical forests are generally seen as niche assembled (Oliveira et al., 2009 and references herein), due to the repeatedly recorded relationship between species composition and the height zone that they occupy along the vertical micro environmental gradient on the trunk of the host trees (Acebey, Gradstein & Krömer, 2003; Cornelissen & ter Steege, 1989; Holz & Gradstein, 2005; Sporn et al., 2010; Wolf, 1994). As poikilohydric plants, they are heavily subjected to the water and light availability gradient along the host trees: temperature, light and wind speed tend to increase towards the canopy, while relative humidity and substrate age tend to decrease (Leon-Vargas, Engwald & Proctor, 2006; Longman & Jeník, 1987; Madigosky, 2004 for description of the general patterns). Still, only a few studies investigated species features that could be related to the establishment of the vertical gradient. In one of the first experiments in this field, osmotic values of leaf cells were shown to determine survival time at low humidity, and correlated to the vertical range of species on their host trees (Hosokawa & Kubota, 1957). Since then, accumulated evidence indicates that species’ ability to withstand desiccation, as well as the relationship between desiccation tolerance and growth form, play a role on species’ habitat specialization along the vertical gradient (Leon-Vargas, Engwald & Proctor, 2006; Pardow & Lakatos, 2013; Proctor, 2002). But most of the species are not habitat specialists, and differences among communities are much more due to variation in species abundances than to species turn over. Moving forward in this field includes the investigation of additional traits as well as the use of species abundances as an indication of more subtle differences along the vertical gradient.

A number of morphological traits have been suggested to influence the interaction of bryophytes with the microclimatic conditions to which they are subjected. For instance, the presence of dark pigmentation is supposed to protect against damaging light intensity (Deltoro et al., 1998), which would facilitate growth in the canopy and be therefore overrepresented in those communities. Observations suggest that deeply-pigmented plants in the maritime Antarctic typically occur in unshaded habitats at the southern end of the species’ range (Newsham & Robinson, 2009). Other traits that may offer advantages to the drier canopy condition are the possession of thickened cell walls, which diminishes water loss, and the ability to convolute leaves, which creates some shade and delays drying (Gradstein, 1994; Schuster, 1983).

Along with the microclimatic conditions of the different height zones, there is also a difference in substrate age and dynamics. The canopy is not only the most recent substrate but also the most frequently subjected to a shortened life span compared to the understory height zones, because branches fall off seven times more often than the tree itself (Marvin & Asner, 2016). If one assumes that the more often species must disperse to new patches, the better must be its dispersal efficiency (Söderström & During, 2005), characters enhancing dispersal efficiency will offer an advantage to canopy colonization. According to the literature, at least two reproductive patterns are associated with higher dispersal ability; (a) monoicy, due to the more frequent sporophyte production in monoicous species when compared to dioicous ones (Batista, Pôrto & Santos, 2018; Longton, 1992; Maciel-Silva, Marques Valio & Rydin, 2012; Oliveira & Pôrto, 1998), which in turn enhance dispersal frequency (but not necessarily distance, see Laenen et al., 2016); (b) production of asexual propagules, due to the advantage of early and abundant diaspore production, especially for within patch dynamics (Söderström, 1989). Apart from the two patterns mentioned, facultative epiphyllous species, subjected to the substrate with the shortest life span in the tropics, should also show relatively high dispersal efficiency (During, 1992), supported by high fertilization success (Alvarenga, Pôrto & Zartman, 2013).

In this study it is assumed that, if a given feature either facilitate or hamper the occurrence of species on a given height zone—independent of the process involved—its frequency will be significantly different than the null expectation for that height zone, generated by a random draw of individuals from the metacommunity. Based on this species sorting perspective, I hypothesize that the species traits mentioned above have a significant role in species assembly of Amazonian epiphytic bryophyte communities along the vertical gradient, being overrepresented in canopy communities. In order to be able to score presence/absence of the same character in all species included in the analysis, as well as to have enough species represented in all height zones, corresponding to the complete microenvironmental gradient, I have chosen the richest and most abundant Amazonian bryophyte family, Lejeuneaceae Cas.-Gil. Lejeuneaceae is the only monophyletic group of epiphytic bryophytes that attend to the above mentioned criteria, including understory specialists, generalists, and canopy specialists (Oliveira & ter Steege, 2013).

## Materials & Methods

The delimitation of the metacommunity used for this study is based on a list of Amazonian bryophyte species growing as epiphytes from a basin wide systematic sampling (Oliveira, 2010). Data includes the species abundance distribution of the complete dataset, i.e., the metacommunity, as well as species abundance distribution of communities of five height zones on the host tree (Oliveira & ter Steege, 2015), well spread over a vertical environmental gradient from the understory to the outer canopy. Species list was updated following the latest taxonomic changes ( Söderström et al., 2016). From the species list, a species character matrix was built for 104 species of Lejeuneaceae, with records of the following: presence of dark pigmentation, ability to convolute leaves when dry, possession of thickened cell walls, reproduction mode (monoicous or dioicous), occurrence of asexual reproduction and epiphyllous habit. All traits were scored as present (1) or absent (0) at species level, and reproductive system as monoicous (1) or dioicous (0) (or both, 0-1, in four species), based mainly on taxonomic literature (Bastos, 2008; Bastos, 2017; Bernecker-Lücking, 1998; Bischler, 1967; Bischler, 1969; Dauphin, 2003; Gradstein, 1994; Gradstein, 1997; Gradstein & Costa, 2003; He, 1999; Ilkiu-Borges & Gradstein, 2008; Ilkiu-Borges & Lisboa, 2004; Jovet-Ast, 1953; Piippo, 1986; Reiner-Drehwald, 1995; Reiner-Drehwald, 2009; Reiner-Drehwald & Goda, 2000; Schuster, 1980). A few cases interpreted as doubtful by the author were scored with an X, and the species was excluded from the analysis of that trait.

The correlation between traits was tested with Principal Components Analysis, in order to exclude this possibility, and found to be not significant. The test whether the occurrence of a given trait on a given height zone was significantly higher or lower than the null expectation was carried out with an R script containing the following instructions: from the species abundance distribution of the complete dataset, 100 random sets were sampled, with the same number of individuals of the real communities per height zone. Average and standard deviation occurrence of each selected trait in the random communities were compared to the ones of the real communities, per height zone. Significant deviations were interpreted as over- or under- representation of the tested trait.

## Results

**Figure 1:**
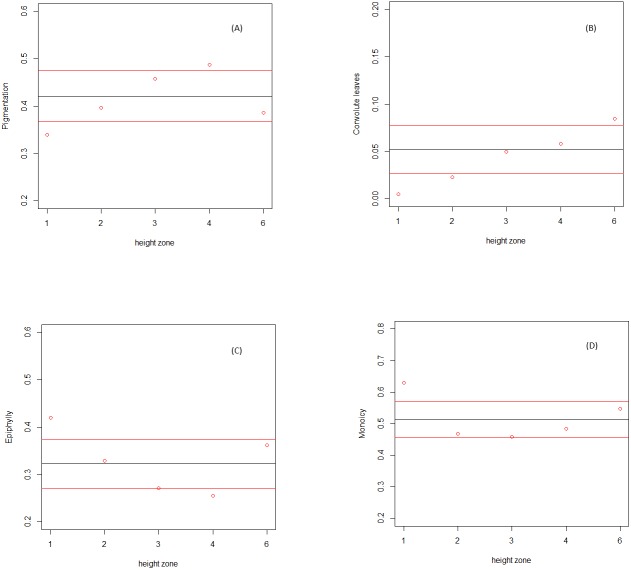
Relative abundance of Lejeuneaceae species traits along the height zones on the host trees. Relative abundance of species traits (*y* axis) along the height zones (*x* axis). Calculated average in field community data (red dots), and calculated average (black line) and standard deviation (red lines) in randomized communities. (A) Pigmentation. (B) Convolute leaves. (C) Facultative epiphylly. (D) Monoicy.

Trait information could be extracted from literature in most of the cases; the coding for convolute leaves and cell wall was omitted in two species each (Table 1). Four out of the six traits tested showed significant higher or lower occurrence in the species composition of a number of height zones: dark pigmentation, convolute leaves, epiphyllous habit and monoicous reproductive mode (Fig. 1). The other two traits—thickening of the cell wall and production of asexual propagules—were found to match percentages of randomly assembled local communities across the complete gradient.

**Table 1:** Species list of Amazonian epiphytic Lejeuneaceae with species relative abundances and character states of the six selected species traits. List of 104 species of Lejeuneaceae recorded in the standardized sampling of bryophyte communities in different height zones of host trees in Amazonian terra firme forests. Species relative abundance in the community (SRA) is taken from field data. Per species, the following character states were compiled from the literature: Sexual system (SEXSY); Production of asexual propagule (APROP); Dark pigmentation (PIGMT); Facultative epiphyllous habit (EPIPH); Leaves convolute when dry (CODRY); Cell wall thickening (CWALL). Coding 0-1 in SEXSY indicates that the species presents both monoicous and dioicous forms, coding X in CODRY and CWALL indicates conflict in the literature or that information available did not fit the binary coding, as interpreted by the author.

Species name	SRA	SEXSY	APROP	PIGMT	EPIPH	CODRY	CWALL
Acrolejeunea emergens (Mitt.) Steph.	2	1	1	0	0	1	1
Acrolejeunea torulosa (Lehm. et Lindenb.) Schiffn.	8	1	1	0	0	1	1
Archilejeunea crispistipula (Spruce) Steph.	14	0	0	0	0	0	1
Archilejeunea fuscencens (Lehm. & Lindenb.) Fulford	70	0	0	0	0	0	1
Brachiolejeunea conduplicata (Steph.) Gradst.	1	1	0	0	0	1	1
Caudalejeunea lehmanniana (Gottsche) A. Evans	16	1	1	0	1	0	1
Ceratolejeunea ceratantha (Nees et Mont.) Schiffn.	2	1	0	1	0	0	1
Ceratolejeunea coarina (Gottsche) Schiffn.	3	1	1	1	1	0	0
Ceratolejeunea confusa R.M. Schust.	8	1	0	1	0	0	1
Ceratolejeunea cornuta (Lindenb.) Steph.	97	1	1	1	1	0	0
Ceratolejeunea cubensis (Mont.) Schiffn.	31	1	0	1	1	0	1
Ceratolejeunea desciscens (Sande Lac.) Schiffn.	2	1	1	1	1	0	1
Ceratolejeunea guianensis (Nees et Mont.) Steph.	31	1	1	1	0	0	0
Ceratolejeunea laetefusca (Austin) R.M. Schust.	48	1	1	1	0	0	0
Ceratolejeunea malleigera (Spruce) Steph.	2	1	1	1	1	0	0
Ceratolejeunea minuta G. Dauphin	26	0	1	1	1	0	1
Cheilojeunea acutangula (Nees) Grolle	2	1	1	0	0	0	0
Cheilojeunea adnata (Kunze ex Lehm.) Grolle	28	0	1	1	0	0	0
Cheilojeunea aneogyna (Spruce) A. Evans	25	1	1	0	0	0	1
Cheilojeunea clausa (Nees et Mont.) R.M. Schust.	16	0	1	0	0	0	X
Cheilojeunea discoidea (Lehm. et Lindenb.) R.M. Schust. et Kachroo	1	1	0	0	0	0	1
Cheilojeunea holostipa (Spruce) Grolle & R.L. Zhu	50	0	0	0	0	0	0
Cheilojeunea neblinensis Ilk.-Borg. & Gradst.	37	0	0	0	0	0	1
Cheilojeunea oncophylla (Ångstr.) Grolle & M.E. Reiner	2	1	1	0	0	0	1
Cheilojeunea rigidula (Nees ex Mont.) R.M. Schust.	148	0	1	0	0	0	0
Cheilojeunea trifaria (Reinw., Blume et Nees) Mizut.	41	1	0	0	0	0	1
Cheilolejeunea unciloba (Lindenb.) Malombe	3	1	0	0	0	0	0
Cheilolejeunea urubuensis (Zartman et I.L. Ackerman) R.L. Zhu et Y.M. Wei	6	1	0	0	0	0	0
Cololejeunea camillii (Lehm.) A. Evans	9	0	1	0	1	0	0
Cololejeunea cardiocarpa (Mont.) A. Evans	1	1	1	1	1	0	0
Cololejeunea contractiloba A. Evans	9	1	1	0	0	0	0
Cololejeunea microscopica (Taylor) Schiffn.	1	1	1	0	1	0	0
Cololejeunea papilliloba (Steph.) Steph.	1	1	1	0	1	0	0
Colura cylindrica Herzog	1	1	1	0	1	0	0
Colura greig-smithii Jovet-Ast	6	0	0	0	1	0	0
Colura tortifolia (Nees et Mont.) Trevis.	1	0	1	0	1	0	1
Cyclolejeuna peruviana (Lehm. et Lindenb.) A. Evans	1	0	1	0	1	0	0
Cyclolejeunea convexistipa (Lehm. et Lindenb.) A. Evans	8	0	1	0	1	0	0
Cyclolejeunea luteola (Spruce) Grolle	5	0	1	0	1	0	X
Dibrachiella auberiana (Mont.) X.Q. Shi, R.L. Zhu & Gradst.	1	1	0	0	0	0	1
Dibrachiella parviflora (Nees) X.Q. Shi, R.L. Zhu & Gradst.	34	1	0	0	0	0	1
Diplasiolejeunea brunnea Steph.	10	0	1	0	1	0	0
Diplasiolejeunea cavifolia Steph.	6	1	1	0	1	0	0
Diplasiolejeunea cobrensis Steph.	5	1	0	0	0	0	0
Diplasiolejeunea pellucida (C.F.W. Meissn. ex Spreng.) Schiffn.	8	0	1	0	1	0	0
Diplasiolejeunea rudolphiana Steph.	18	1	1	0	0	0	1
Drepanolejeunea crucianella (Taylor) A. Evans	2	0	1	0	1	0	1
Drepanolejeunea fragilis Bischl. ex L. Söderstr., A. Hagborg et von Konrat	30	0	1	0	0	0	1
Drepanolejeunea lichenicola (Spruce) Steph.	2	0	1	0	1	0	1
Drepanolejeunea orthophylla (Nees et Mont.) Bischl.	3	0	1	0	1	0	0
Frullanoides liebmanniana (Lindenb. et Gottsche) van Slageren	1	0	0	1	0	1	1
Haplolejeunea amazonica Ilkiu-Borges & Gradst.	14	1	0	0	0	0	0
Harpalejeunea oxyphylla (Nees et Mont.) Steph.	19	0	0	1	1	0	0
Harpalejeunea stricta (Lindenb. et Gottsche) Steph.	10	0	0	0	0	0	0
Harpalejeunea tridens (Besch. et Spruce) Steph.	3	0	0	0	1	0	0
Lejeunea adpressa Nees	15	1	0	0	1	0	0
Lejeunea asperrima Spruce	2	1	1	0	1	0	0
Lejeunea asthenica Spruce	1	1	0	0	1	0	1
Lejeunea boryana Mont.	2	1	1	0	1	0	1
Lejeunea cerina (Lehm. et Lindenb.) Lehm. et Lindenb.	7	0	1	0	0	0	0
Lejeunea controversa Gottsche	11	1	0	0	0	0	0
Lejeunea deplanata Nees	1	0	1	0	1	0	0
Lejeunea flava (Sw.) Nees	16	1	0	0	1	0	0
Lejeunea laetevirens Nees et Mont.	44	0	1	0	1	0	0
Lejeunea monimiae (Steph.) Steph.	1	1	0	0	1	0	0
Lejeunea obtusangula Spruce	1	1	0	0	1	0	0
Lejeunea phyllobola Nees et Mont.	18	1	1	1	0	0	1
Lejeunea reflexistipula (Lehm. et Lindenb.) Lehm. et Lindenb.	14	0	0	1	0	0	1
Lejeunea saccatiloba (Steph.) R.L. Zhu & W. Ye	3	0	0	1	0	0	0
Lepidolejeunea cordifissa (Taylor) M.E. Reiner	1	0	0	0	1	0	0
Lepidolejeunea involuta (Gottsche) Grolle.	10	0	1	0	1	X	0
Leptolejeunea elliptica (Lehm. et Lindenb.) Besch.	21	0-1	1	0	1	0	1
Leptolejeunea obfuscata (Spruce) Steph.	2	0	0	1	1	0	1
Lopholejeuna eulopha (Taylor) Schiffn.	3	1	1	1	0	0	1
Lopholejeunea nigricans (Lindenb.) Schiffn.	2	0-1	0	1	0	0	1
Lopholejeunea subfusca (Nees) Schiffn.	56	1	0	1	0	0	1
Macrocolura sagittistipula (Spruce) R.M. Schust.	5	1	1	0	0	0	1
Metalejeunea cucullata (Reinw., Blume et Nees) Grolle	1	1	0	0	1	0	1
Microlejeunea acutifolia Steph.	17	0	1	0	0	0	1
Microlejeunea bullata (Taylor) Steph.	36	0	1	0	0	0	1
Microlejeunea epiphylla Bischl.	24	0	1	0	1	0	0
Microlejeunea globosa (Spruce) Steph.	5	0	1	0	0	0	0
Neurolejeunea breutelii (Gottsche) A. Evans	8	0	1	1	0	X	1
Neurolejeunea seminervis (Spruce) Schiffn.	8	0	0	1	0	0	1
Odontolejeunea lunulata (F. Weber) Schiffn.	1	1	1	0	1	1	0
Odontolejeunea rhomalea (Spruce) Steph.	2	0	1	0	1	1	0
Pictolejeuna picta (Steph.) Grolle	22	1	1	0	0	0	0
Priolejeunea aemula (Gottsche) A. Evans.	5	1	1	0	1	0	0
Prionolejeunea denticulata (F. Weber) Schiffn.	6	1	1	0	1	0	0
Prionolejeunea muricatoserrulata (Spruce) Steph.	7	1	1	0	1	0	0
Pycnolejeunea contigua (Nees) Grolle.	27	1	1	1	0	0	1
Pycnolejeunea macroloba (Nees et Mont.) Schiffn.	52	0-1	1	1	0	0	1
Pycnolejeunea papillosa Xiao L. He	4	1	1	1	0	0	1
Rectolejeunea emarginuliflora (Schiffn.) A. Evans	14	0	1	1	0	0	0
Rectolejeunea flagelliformis A. Evans	26	1	1	0	0	0	0
Rectolejeunea versifolia (Schiffn.) L. Söderstr. et A. Hagborg	11	0	1	0	1	0	0
Stictolejeunea squamata (Willd.) Schiffn.	25	0	0	1	1	0	1
Symbiezidium barbiflorum (Lindenb. et Gottsche) A. Evans.	55	1	0	1	1	0	1
Symbiezidium transversale (Sw.) Trevis.	25	0-1	0	1	0	0	1
Thysananthus amazonicus (Spruce) Schiffn.	17	1	0	1	0	1	0
Thysananthus auriculatus (Wilson & Hook.) Sukkharak & Gradst.	42	1	0	1	0	1	0
Verdoonianthus griffinii Gradst.	10	1	0	0	0	1	1
Vitalianthus aphanellus (Spruce) Bechteler	2	1	0	0	0	0	1
Xylolejeunea crenata (Nees et Mont.) Xiao L. He et Grolle	5	1	1	1	1	0	0

Dark pigmentation was found to be significantly more represented in the bifurcation zone (zone 4), but not in the outer canopy (zone 6) or along the trunk (zones 2 and 3). The occurrence of this trait in communities at the base of the tree (zone 1) was significantly lower than expected by chance. Convolute leaves were significantly more represented in the outer canopy (zone 6), and significantly less in the first two understory zones (zones 1 and 2). Both the monoicous reproduction system and the facultative epiphylly were significantly more represented only in communities at the base of the tree (zone 1), with the latest significantly less represented only in communities in the bifurcation zone.

## Discussion

For bryophytes, the vertical gradient along the host trees reflects the two extremes of a water balance axis: in the canopy, plants may die from desiccation due to evaporation and to the unavailability of water for photosynthesis and growth; in the understory, from the lack of enough light to achieve net carbon gain, given the relatively high temperature. Interestingly, in this study, the traits supposed to protect against the harsh conditions of the canopy were not only significantly more frequent there, but showed also significantly lower occurrence in the darkest zone of the understory, the tree base.

Bryophytes show light saturation of photosynthesis at modest irradiance when compared to most vascular plants (Marschall & Proctor, 2004), and light may be a threat, especially in the case of associated water loss. In the absence of a waxy cuticle or stomata—present only in the sporophyte of mosses and hornworts—bryophytes can dry out very quickly. Also exposure to strong light was shown to decrease photosynthetic rates in *Sphagnum* species due to photoinibition (Harley et al., 1989). It was therefore expected that the presence of dark pigmentation, which may help avoiding the damage that higher UV- radiation causes to the photosystem, had a significantly higher abundance in the canopy. However, it is difficult to explain why the feature was specifically related to the inner canopy (zone 4), but not to the outer canopy communities (zone 6), which are even more exposed to sun radiation. One explanation may be that a trait not included in this study, has an additional influence on species’ response to radiation. For instance, levels of methanol extractable UV-absorbing compounds (MEUVAC) in response to high UV radiation were shown to be higher in liverworts than in mosses (Arróniz-Crespo et al., 2004), but they may also vary among liverwort taxa. Convolute leaves, significantly more present in outer canopy communities, increase self-shade and help retaining the external capillary water, one of the strategies of bryophytes to remain hydrated (Proctor et al., 1998).

In the Amazonian lowlands, low light levels during the day combined with moist and warm conditions at night promotes high respiration rates, which in turn causes a limitation of net carbon gain (Bader et al., 2013; Wagner et al., 2013). That is probably the best explanation why the same traits offering protection from high light intensity or promoting water retention—pigmentation and convolute leaves, are the ones occurring significantly lower than expected in the understory. In the understory conditions, close to the forest floor, species should either optimize light capture to keep net carbon gain or to be able to dry fast, stopping respiration losses. That means that these traits are not only unnecessary, they actually stop most of the individuals of the species possessing it from growing in the understory.

The reproductive traits supposed to be relevant for the assembly of canopy communities met the expectations poorly, which could be a shortcoming of the data, because the characters treated in this study are only indirectly related to high dispersal ability. Still, I believe that a better explanation for the results obtained is that dispersal features have simply little influence on community assemblage. This relatively less deterministic role of dispersal in bryophyte assemblages has been supported especially by recent studies with a mechanistic approach of species assemblage that take into account the relationship between metacommunity and local communities (Barbé, Fenton & Bergeron, 2016; Udd et al., 2015). For instance, the ability to produce asexual propagules, claimed as a dispersal advantage and here hypothesized to be related to the dynamic canopy microenvironment, was not significantly associated to any height zone. Although the character seems to be ecologically and evolutionary related to epiphylly (Kraichak, 2012), the latest showed slightly different variation, being overrepresented in the understory. Therefore, the results suggest that the expected role of epiphylly in the outer canopy assemblage occupation (Oliveira & ter Steege, 2013), is not necessarily due to high dispersal ability given by asexual reproduction, as expected, but perhaps to the ability of facultative epiphylls to adhere to smooth surfaces (Cornelissen & ter Steege, 1989; Gradstein, 1997). The significantly higher facultative epiphylly on trunk bases (zone 1) might be related to the proximity of populations of these same species on the leaves of understory shrubs (Zartman & Ilkiu-Borges, 2007), which increases the chance of colonization.

Production of asexual propagules and dioicous reproductive mode are frequently taken as associated features (Löbel, Snäll & Rydin, 2009), and even used as a trade-off relationship on geographical distribution. In the epiphytic Lejeuneaceae species studied, they were not significantly related. Also the result obtained for reproductive system seemed counterintuitive. Monoicy, as a surrogate for relatively frequent spore production, was significantly overrepresented only at the tree base, instead of in the canopy, as found among liverworts in French Guiana (Gradstein, 2006).

Several relevant traits that play a role on plant dispersal, establishment and growth are not represented by presence/absence data. For instance, the responses of photosynthesis to irradiance of epiphytic bryophytes show compensation points over a wide range among species (Gabriel & Bates, 2003), as much as the photosynthetic capacity after a desiccation event also varies among species (Deltoro et al., 1998; Johnson & Kokila, 1970; Pardow & Lakatos, 2013). In particular, physiological and hydrology-related traits can be analysed and compared through continuous variables, provided that measurements follow a standard protocol that captures their variation in a comparable manner (Cornelissen et al., 2007). The presence of thickening on the cell walls, which may allow liverworts to hold water relatively longer inside the cells, extending photosynthetic activity, showed no significant results in this study. Probably its relevance was not detectable because information was compiled as a binary character rather than a continuous one. The variation on the thickening, or the proportion thickness/cell lumen probably does matter for the water balance of the species along the gradient. Further examples of continuous characters of Lejeuneaceae that may have an influence on water balance—also with the possibility of being damaging under low light conditions—include: the rate of leaf lobule to leaf lobe, the degree of leaf imbrication, and the reduction of lobules, can also show intraspecific variation (Gradstein, 1994; Schuster, 1983). Furthermore, dispersal traits also deserve more direct approach, which takes into account differences in phenology and spore features.

## Conclusions

The presence of dark pigmentation and the presence of convolute leaves seem to have a relevant influence on the occurrence of species at both extremes of the forest vertical microenvironmantal gradient, either favouring—in the canopy—or hampering—at the base of the tree—the number of individuals assembled in the communities. Species traits related to morphological features showed greater influence on the occurrence of species than traits related to reproduction and dispersal. Further advances in this field will profit from the study of traits with continuous variation, such as the ones mentioned in the discussion.

## Additional Information and Declarations

### Data Availability

The following information was supplied regarding data availability: Raw data used for all data analysis are presented in Table 1.
